# Current-driven dynamics of skyrmions stabilized in MnSi nanowires revealed by topological Hall effect

**DOI:** 10.1038/ncomms9217

**Published:** 2015-09-24

**Authors:** Dong Liang, John P. DeGrave, Matthew J. Stolt, Yoshinori Tokura, Song Jin

**Affiliations:** 1 Department of Chemistry, University of Wisconsin-Madison, Madison, Wisconsin 63706, USA; 2 RIKEN Center for Emergent Matter Science (CEMS), Wako 351-0198, Japan; 3 Department of Applied Physics and Quantum-Phase Electronics Center (QPEC), University of Tokyo, Tokyo 113-8656, Japan

## Abstract

Skyrmions hold promise for next-generation magnetic storage as their nanoscale dimensions may enable high information storage density and their low threshold for current-driven motion may enable ultra-low energy consumption. Skyrmion-hosting nanowires not only serve as a natural platform for magnetic racetrack memory devices but also stabilize skyrmions. Here we use the topological Hall effect (THE) to study phase stability and current-driven dynamics of skyrmions in MnSi nanowires. THE is observed in an extended magnetic field-temperature window (15–30 K), suggesting stabilization of skyrmions in nanowires compared with the bulk. Furthermore, we show in nanowires that under the high current density of 10^8^–10^9^ A m^−2^, the THE decreases with increasing current densities, which demonstrates the current-driven motion of skyrmions generating the emergent electric field in the extended skyrmion phase region. These results open up the exploration of skyrmions in nanowires for fundamental physics and magnetic storage technologies.

## Abstract (web summary)

Magnetic skyrmions are topologically protected magnetization textures which can arise in helical magnets and present promise for low-power nanoscale magnetic storage device applications. Here, the authors demonstrate extended phase stability and current-driven dynamics of skyrmions in nanowires of MnSi.

## Graphical abstract

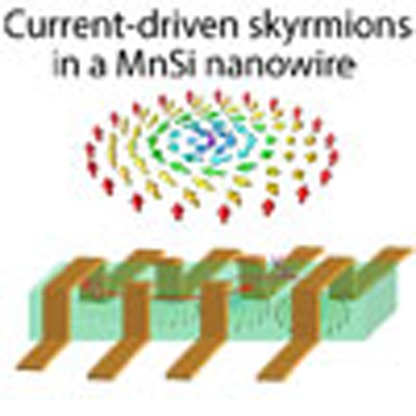

A magnetic skyrmion is a vortex-like spin texture in which the central spin is anti-aligned with the externally applied magnetic field and the outer spins are aligned^1^,^2^. Their formation results from the competition of the ferromagnetic exchange and the Dzyaloshinskii–Moriya interaction that appears in crystal systems lacking a center of inversion symmetry, such as the non-centrosymmetric cubic B20 crystal system, including MnSi (refs 3, 4, 5), Fe_1-*x*_Co_*x*_Si (ref. 6), FeGe (ref. 7), MnGe (ref. 8). Skyrmion domains can be manipulated using remarkably low current densities compared with ferromagnetic domain walls, which has become the focus of intense interest for future low-power consumption nanoscale spintronic devices^2^,^9^,^10^,^11^,^12^. The relative stability and ease of manipulating magnetic skyrmions with the electrical current result from their non-trivial topology, smoothly varying spin configuration and unique ability to deform and avoid pinning sites^2^,^13^. This ultra-low current density may enable low-power consumption applications, and help to avoid common failure modes associated with very large current densities encountered in ferromagnetic systems being explored for magnetic racetrack memory concepts^14^.

To explore skyrmions for potential magnetic storage technology, we need to study the stability and dynamics of skyrmions in skyrmion-hosting nanoscale systems^12^,^15^,^16^. In particular, nanowires (NWs) not only serve as a natural platform for magnetic racetrack memory devices^14^ using skyrmions as information units, but also could have significant advantages over bulk materials to stabilize skyrmions^17^,^18^. For example, as the dimension of the system approaches the size of the helical pitch that characterizes the helimagnetic ground state, the conical magnetic configuration will be destabilized energetically compared with the skyrmion state^17^. This has been experimentally observed in thin films of B20 materials with nanoscale thickness^5^,^7^,^19^,^20^. The stabilization of the skyrmions was also theoretically predicted in nanoscale systems by the introduction of large anisotropy energy^18^. Furthermore, anisotropic NWs with small cross-sections are natural platforms for studying emergent electrodynamics associated with current-driven skyrmions at much larger current densities than can ever be achieved in bulk. Therefore, NW systems can be used to manipulate skyrmions on faster timescales and study the fundamental dynamics of the skyrmions^2^,^15^,^16^.

Skyrmions and their current-driven motion in B20 crystals have been studied by reciprocal space observation using small-angle neutron scattering in bulk crystals of MnSi (ref. 9) and by real space observation using Lorentz transmission electron microscopy (LTEM) in FeGe thin films^10^. These experiments have shown that the skyrmion domains translate primarily along the current direction at a current density on the order of 10^6^ A m^−2^, which is up to 4–5 orders of magnitude smaller than the current densities required for the translation of ferromagnetic and helimagnetic domain walls^13^,^21^. Even though stabilization of the skyrmion lattice in MnSi NWs has been suggested using LTEM observations of a thinned NW slab^5^, magnetoresistance^22^, the dynamics of skyrmions in NW systems is more difficult to investigate. Hall transport measurements, especially the observations of topological Hall effect (THE) beyond the normal Hall effect (NHE) and anomalous Hall effect (AHE), have been considered as an electrical transport signature of skyrmions^2^,^11^,^19^,^20^,^23^,^24^. This is because when a conduction electron passes through a topologically non-trivial spin texture, the spin of the conduction electron adiabatically couples to the local spin and acquires a quantum-mechanical Berry phase that can be re-formulated in terms of an effective magnetic field^2^,^11^, which deflects the conduction electrons perpendicular to the current direction. Therefore, the presence of skyrmions will cause an additional contribution to the observed Hall signal that has been termed THE. Furthermore, when the skyrmions start to move above the critical current density, the THE effect is predicted^25^ and observed^11^ to decrease due to suppression of the THE by the emergent electric field. If the skyrmion domains are to be exploited for magnetic storage applications, emergent electrodynamics study of the current-driven skyrmion motion need to be extended to nanoscale geometries, which allow much higher current densities and potentially higher velocities of the skyrmion motion to be studied^13^. Therefore, despite the challenging NW geometry for fabricating Hall devices^26^, there exists a strong motivation to investigate skyrmion dymanics of NWs using the THE.

Here, using Hall effect measurements of MnSi NWs, we report the observation of the THE due to the stabilized skyrmions and the study of their current-driven dynamics at large current densities (10^8^–10^9^ A m^−2^). We clearly demonstrate the extraction of the topological portion of the Hall signal from the total Hall signal and use the THE to construct magnetic field (*B*)-temperature (*T*) phase diagrams for MnSi NWs, which show that skyrmions are stable in these MnSi NWs over a larger *B*–*T* range. Furthermore, for the first time, we study the electrodynamics of current-driven skyrmions in NW morphology at large current densities and show the suppression of the THE due to the emergent electric field arising from current-driven motion of skyrmions and estimate the skyrmion drift velocity.

## Results

### THE of a single MnSi NW

**Figure 1:**
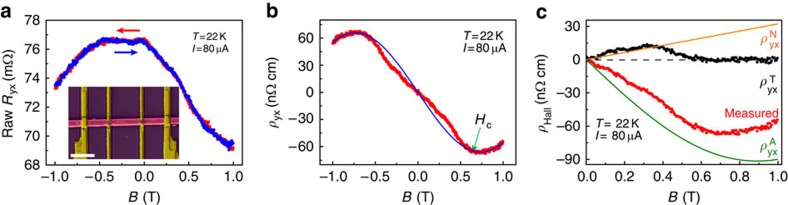
Extraction of the THE resistivity 
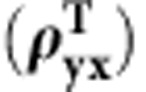
 of NW1 (330 nm wide by 270 nm thick). (**a**) The raw Hall resistances shown as red (positive field sweep from −1 to 1 T) and blue (negative field sweep from 1 to −1 T) curves measured between the transverse Hall electrodes at *T*=22 K and *I*=80 μA (current density *j*=9.16 × 10^8^ A m^−2^). Inset shows a typical NW device with classical Hall bar. The scale bar is 2 μm. (**b**) The antisymmetric Hall resistivity (*ρ*_yx_) extracted from the raw Hall resistance in panel **a**. The blue line shows the polynomial fitting above critical field *H*_c_ and extrapolation below *H*_c_ to simulate the normal and anomalous Hall contributions to the total Hall resistivity. (**c**) Extracted topological Hall signal in comparison with normal and anomalous Hall contributions. The topological Hall resistivity 
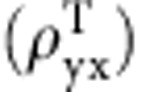
 in black curve is extracted by subtracting the fitting (blue curve in panel **b**) from the total measured Hall signal (red curve in panel **b**). The fitting curve is also decomposed to normal (orange line) and anomalous (green curve) Hall contributions.

The Hall devices were fabricated using single crystal MnSi NWs synthesized by chemical vapour deposition^27^ and using an improved device fabrication strategy, in which three-step angled evaporations of metal electrodes (see Supplementary Fig. 1) ensure side-wall Hall electrode contact^28^, but now the opposing Hall electrodes are aligned as in the classical Hall-bar devices to make the Hall signal stand out. The inset of Fig. 1a shows a typical SEM image of a representative MnSi NW Hall device. Both transverse (Hall) and longitudinal resistance in magnetic field were measured. We will focus the discussion on the data from two representative MnSi NW devices, namely NW1 (330 nm wide by 270 nm thick) and NW2 (shown in Fig. 1a inset, 480 nm wide by 300 nm thick). We only collected data in the magnetic field range from −1 to +1 T, which was determined to be the region of the greatest interest through preliminary scans for identifying the THE attributed to the skyrmion phase in MnSi NWs, and was additionally suggested by previous LTEM observations of a MnSi NW from the same synthesis^5^. Figure 1a show the raw transverse resistance measured between opposing Hall-bar electrodes of NW1 (ref. 28) at *T*=22 K and current *I*=80 μA. The data were obtained by sweeping magnetic field in both positive (−1 to 1 T) and negative (1 to −1 T) directions as indicated by blue and red dots, respectively. The two curves show no significant hysteresis and overlap very well with a very tiny deviation, which is consistent with the behaviours of helical and conical phase. Then we took average of the two curves and antisymmetrized the averaged data to obtain actual Hall resistance (*R*_yx_). After that, the Hall resistivity (*ρ*_yx_), shown as the red curve in Fig. 1b, is calculated by


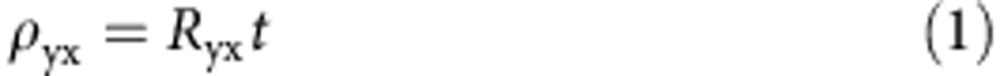


where *t* is the thickness of the NW.

We will use the representative data shown in Fig. 1b to illustrate the procedures for extracting the THE. The total Hall resistivity (*ρ*_yx_) measured in the MnSi NW in the region of the skyrmion phase consists of three components of resistivity,


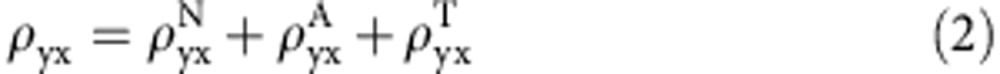


Where the three terms on the right side correspond to the NHE attributed to Lorentz forces on the conduction electrons due to the externally applied magnetic field, the AHE which is known to scale with the magnetization of the sample, and the THE which is attributed to the emergent magnetic fields associated with the skyrmion domains, respectively. Above the critical field (*H*_c_) for the transition between the conical and the field-induced ferromagnetic state, the dominant contribution to the measured Hall signal is the NHE and AHE. The NHE is expressed as 
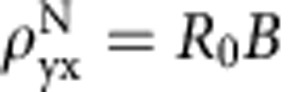
, where *R*_0_ is the normal Hall coefficient. The AHE contribution 
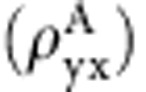
 is expressed as 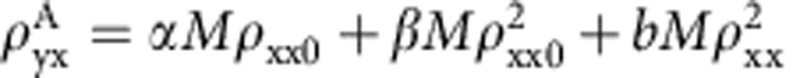 where *α*, *β* and *b* are constants corresponding to the skew scattering, side jump, and intrinsic contributions to the anomalous Hall resistivity, *ρ*_xx0_ is the residual resistivity, and *M* is the magnetization^20^,^29^. The THE can be extracted by subtracting the NHE and AHE from total Hall signal. The linear dependence of AHE on *M* reported for MnSi (ref. 30) allows us to calculate the 
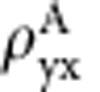
 quantitatively if *M* is measured. However, the direct measurement of *M* in NWs in perpendicular *B* is very challenging. Instead we must rely on a phenomenological assumption for the AHE inferred from previous works on bulk MnSi (ref. 23) and thin films of MnSi (ref. 20). Namely, we assume that the magnetization is smoothly varying in the region of skyrmion phase, together with the fact that AHE and NHE dominate above the critical field (*H*_c_), the AHE and NHE can be simulated by fitting the total Hall effect signal above the critical field (*H*_c_) and extrapolating below *H*_c_, as shown in the blue curve in Fig. 1b. We accomplish this fitting by using a degree-five polynomial function *pB*+*qB*^3^+*rB*^5^ with fitting parameters of *p*, *q*, *r* to represent the NHE and AHE contributions in the total magnetic field of interest. Then we perform the subtraction of simulated NHE and AHE to obtain THE confidently in the suspected region of the skyrmion formation based both on previous transport studies of MnSi (refs 23, 31) as well as recent LTEM results of a thinned MnSi NW specimen^5^. We note that in principle a similar phenomenological data extraction was applied to recent experiments of the THE anomaly in bulk MnSi in which the non-topological Hall signal was fit using the assumption of a linear background^11^,^32^. The extracted THE signal is shown in Fig. 1c as the black curve. The blue fitting curve in Fig. 1b is decomposed to NHE (orange line) and AHE (green curve) components in Fig. 1c. We notice the NHE at all temperatures of interest indicates hole-like carriers, which is consistent with those reported for MnSi bulk^24^,^31^ and thin film^20^. In contrast, the AHE is negative and dominates the Hall signal. The extracted THE has the same sign as the NHE, but opposite sign to the AHE. These observations are consistent with those reported in MnSi thin film^20^.

### Skyrmion phase diagram from THE

**Figure 2:**
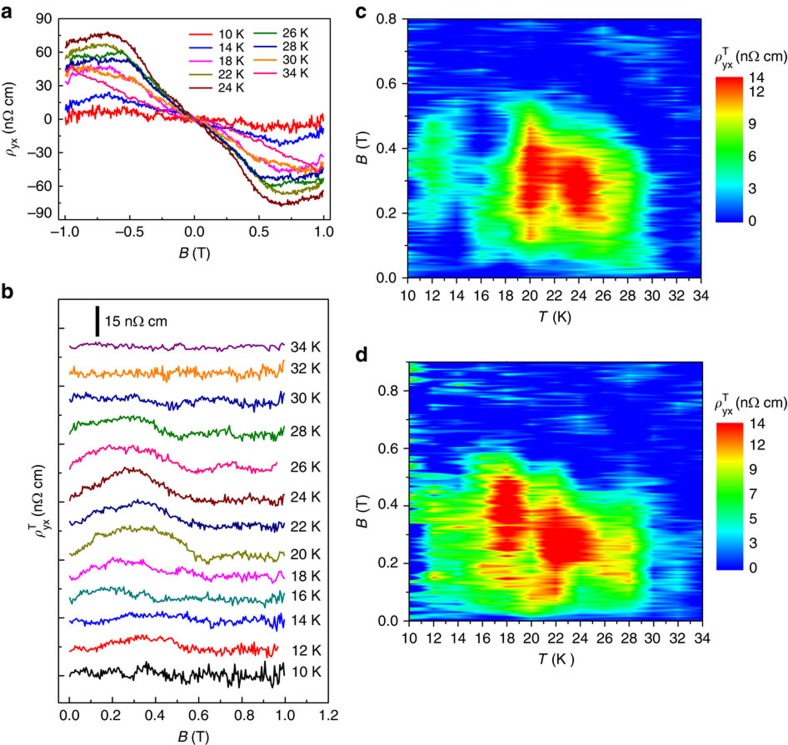
Topological Hall resistivities and the magnetic phase diagrams for two MnSi NW devices. (**a**) Hall resistivity from antisymmetrization of raw data from 10 to 34 K and (**b**) Topological Hall resistivity 
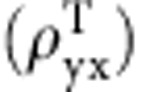
 for NW1 (330 nm wide by 270 nm thick) at several temperatures extracted using the polynomial fitting procedure detailed in the text. (**c**,**d**) Phase diagrams of extracted 
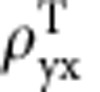
 as a function of the external magnetic field (*B*) and temperature (*T*) for NW1 and NW2 (480 nm wide by 230 nm thick) constructed by interpolating 
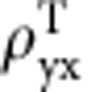
 between temperatures, respectively. All data are collected at *I*=80 μA, corresponding to a current density of 9.16 × 10^8^ A m^−2^ for NW1 and 5.52 × 10^8^ A m^−2^ for NW2.

Hall resistivities at various temperatures from 10 to 34 K are plotted in Fig. 2a. We then extracted the topological Hall resistivity 
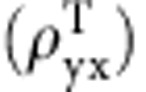
 via subtracting the polynomial fitting from *ρ*_yx_ at various temperatures, as discussed above. The extracted 
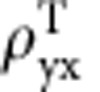
 values for NW1 (330 nm wide by 270 nm thick) from 10 to 34 K with the step of 2 K are shown in Fig. 2b. The magnitude of the THE 
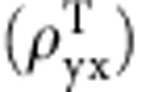
 is ∼15 nΩ cm at 22 K, slightly larger than that measured in MnSi bulk^11^,^23^ and thin film^20^. We further use this set of data to construct the interpolated pseudo-color *B*–*T* phase diagram shown in Fig. 2c. To show the generality of the topological Hall signal, we performed the same analysis on a second MnSi NW device (NW2 of 480 nm wide and 300 nm thick) and constructed the phase diagram for the 
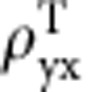
 shown in Fig. 2d. For both NW1 and NW2, the skyrmion phase has been identified in an extended phase region from a minimum of 15 K up to a *T*_c_ of roughly 30 K and a magnetic field region from 0.1 to 0.5 T. Note that the temperatures reported in the phase diagrams (Fig. 2c,d) can be slight underestimates of the true NW device temperatures because of the Joule heating effect, which will be discussed in detail later.

The magnitude of 
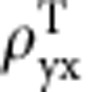
 is known to depend on several factors and can be written as 
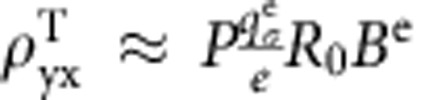
 (ref. 23), where *P* is the spin polarization of charge carriers, 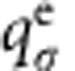 is the emergent charge associated with the charge carrier spins (
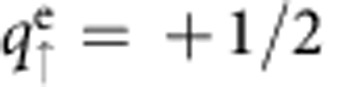
 and 
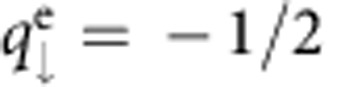
), *e* is the elementary charge, *R*_0_ is the normal Hall coefficient, and *B*^e^ is the emergent magnetic field associated with one unit cell of the skyrmion lattice. *B*^e^ is proportional to the skyrmion winding number 
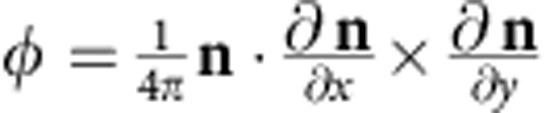
 where 
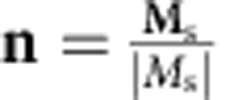
 is the local magnetization direction, and *x* and *y* are the coordinates perpendicular to the applied magnetic field^2^. The value of *B*^e^ is roughly 13.1 T|*e*/*q*^e^| for the hexagonal lattice of skyrmions in MnSi (ref. 23). Here for MnSi NWs, LTEM study^5^ showed no change to the size and density of skyrmions, so we don't expect much change in *B*^e^. However, *R*_0_ in our NWs is about twice as large as that in bulk MnSi and the spin polarization (*P*) might also change in NWs, therefore it is not unexpected that 
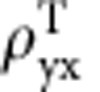
 of magnitude ∼15 nΩ cm is higher than those in bulk^11^,^23^ and thin film^20^. Note that the observed 
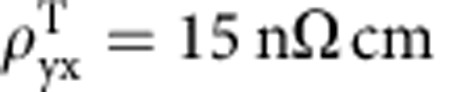
 at 80 μA is not the THE for stationary skyrmions, later it will be shown that at 80 μA skyrmions are in motion.

The temperature window in which we observe the THE signal in MnSi NWs is roughly between 15 and 30 K, which places these MnSi NW samples somewhere between the observed behaviour in bulk MnSi with a narrow skyrmion phase region (26–29.5 K)^23^, and the observed behaviour for strained 50 nm thick MnSi thin films grown by molecular beam epitaxy with a wider skyrmion phase region (2–45 K)^20^. Since the width and thickness of our NW samples on the order of 300 nm are much larger than the skyrmion size of 18 nm in MnSi, spatial confinement effect on skyrmions can be excluded. Uniaxial magnetic anisotropy, from intrinsic strain or surface effect, is more likely to induce the extension of the THE region^18^. In particular, large surface-to-volume ratio of NWs is likely to manifest the surface effect-induced uniaxial anisotropy and hence could explain the extended stabilization of skyrmions in MnSi NWs^5^,^33^. We shall clarify that the THE measurements detect the spin chirality in the system, so the extracted skyrmion phase above can either be skyrmion lattice or possibly be mixture of skyrmions with other magnetic states^7^.

### Anisotropy of skyrmion phase stability

**Figure 3:**
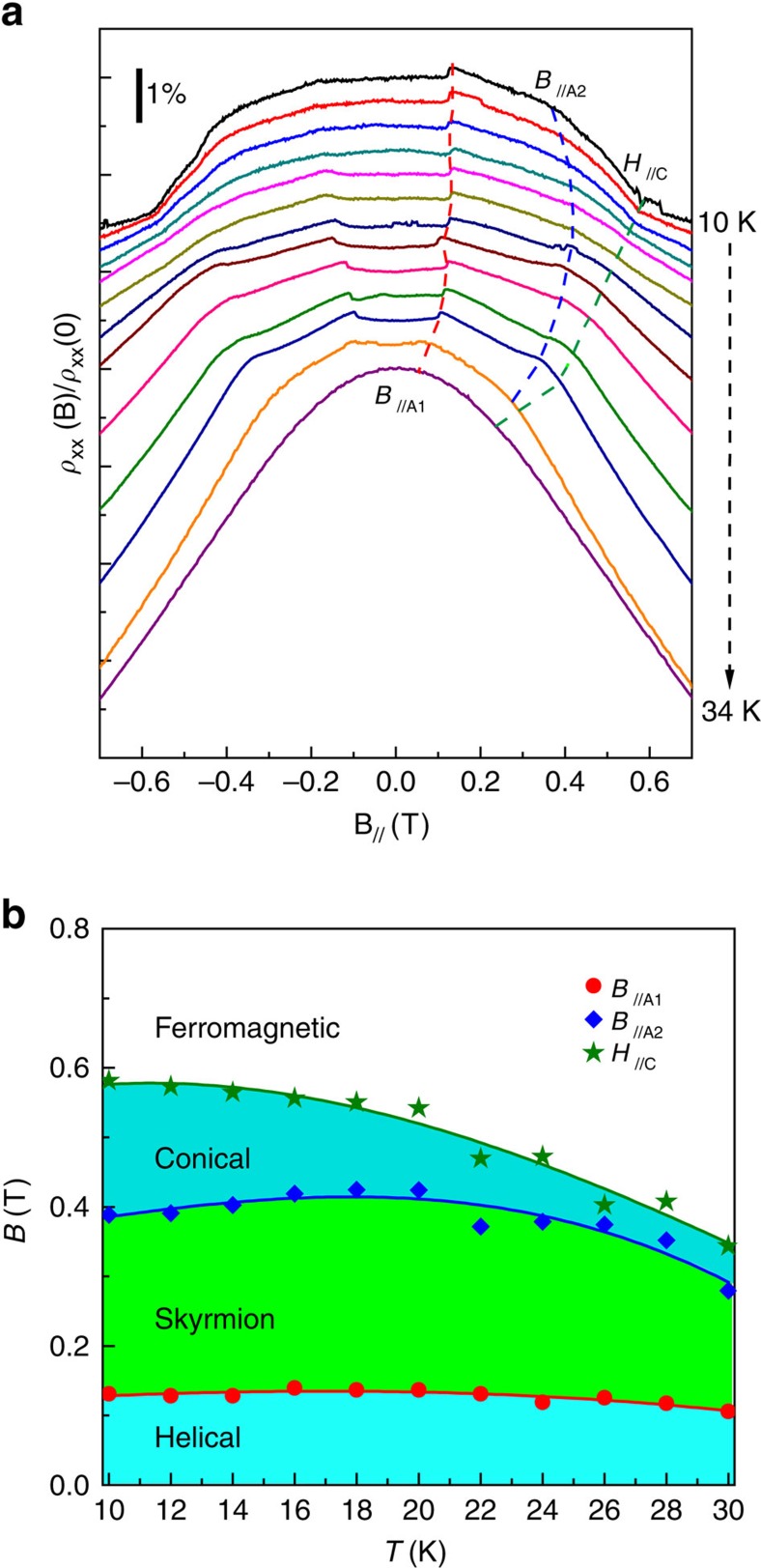
Magnetoresistivity and corresponding magnetic phase diagram of NW1 (330 nm wide by 270 nm thick) in a parallel magnetic field. The externally applied magnetic field is preferentially parallel to the long axis of the NW (<10° misalignment). (**a**) Normalized longitudinal magnetoresistivity measured from 10 to 34 K with a step of 2 K at a current *I*=80 μA (current density *j*=9.16 × 10^8^ A m^−2^). The dash lines indicating the approximate positions of critical fields corresponding to the *B*_*//*A1_ (red filled circles), *B*_*//*A2_ (blue diamonds) and *H*_*//*c_ (green stars) in the *B*–*T* phase diagram in panel **b**. (**b**) Magnetic phase diagram obtained from the longitudinal magnetoresistivity in a parallel magnetic field. The phase boundaries revealed by longitudinal magnetoresistivity are represented by red filled circles (*B*_*//*A1_), blue diamonds (*B*_*//*A2_) and green stars (*H*_//c_), which correspond to transitions from helical to skyrmion states, from skyrmion states to conical states, and from conical to field-polarized ferromagnetic states, respectively. Skyrmion phase is shown to persist from 30 K down to 10 K (the lowest temperature in our measurements).

The magnetic anisotropy in MnSi NWs is further supported by the influence of external magnetic field orientation on the skyrmion phase stability inferred from the THE in perpendicular magnetic field and from the longitudinal magnetoresistivity in parallel field. For the latter measurements, the NW long axis was aligned under an optical microscope within a 10° error before mounting the device chip. This magnetic field orientation allows us to observe the effects of the strong anisotropy from the NW morphology on the formation of the skyrmion phase. Similar to what we have reported^22^, the longitudinal magnetoresistivity of NW1 (330 nm wide by 270 nm thick) in Fig. 3a shows three kinks (highlighted by dash lines) that are clear signatures of three critical fields identified as: *H*_*//*c_ (commonly referred to as *B*_C2_ in MnSi literature) corresponding to the transition field from conical to field-polarized ferromagnetic states; *B*_*//*A2_ corresponding to the transition field from skyrmion to conical states; and *B*_*//*A1_ as the transition field from helical to skyrmion states. These critical fields have been identified in magnetoresistance of MnSi bulk^34^ and NW samples^22^ and have been attributed to the skyrmion phase. The *B*_*//*A1_ (filled circles), *B*_*//*A2_ (diamonds) and *H*_*//*c_ (stars) determined by the kinks in magnetoresistivity of NW1 in a parallel magnetic field are plotted in Fig. 3b. The skyrmion phase indicated by longitudinal magnetoresistivity persists from 30 K down to the lowest measurement temperature 10 K in parallel magnetic field orientation (actually it can go down to 3 K according to our previous report^22^), whereas the skyrmion phase inferred from the THE measurements exist from 15 to 30 K in perpendicular orientation. These differences in skyrmion phase diagrams between the two orientations indicate strong magnetic anisotropy that could enable a positive uniaxial anisotropy to stabilize the THE (and skyrmion) phase, since theory predicts that the uniaxial anisotropy in B20 crystals is essential in stabilization of skyrmions^18^,^19^.

### Current-driven dynamics of skyrmions in a MnSi NW

Furthermore, we studied the current-density dependence of the THE in MnSi NWs. We typically used applied currents of between 40 and 240 μA, which corresponds to the current density regime of 10^8^–10^9^ A m^−2^, depending on the NW cross section dimensions. The maximum current densities applied to NW1 and NW2 were on the order of 10^9^ A m^−2^ (2.75 × 10^9^ A m^−2^ for NW1 and 1.38 × 10^9^ A m^−2^ for NW2), which are roughly three orders of magnitude larger than the critical current density required for depinning the skyrmion lattice observed for high purity bulk crystals of MnSi, yet still much lower than the upper threshold current density (on the order of 10^12^ A m^−2^) beyond which the melting of the skyrmion lattice into a chiral liquid phase is suggested to occur by a recent calculation^35^. Such high current densities enabled by the NW geometry of our samples offer a unique regime to study the skyrmion dynamics.

The side effect of such high current densities is the local Joule heating of the NW device. For currents larger than 40 μA, both devices showed non-ohmic *I*–*V* curves (or increase of resistance) and decrease of critical field *H*_c_, as shown in Supplementary Fig. 2. This indicates that large currents raised the local device temperature to a value higher than the nominal cryostat temperature. Significant Joule heating effect, observed at currents larger than 80 μA, made it difficult to examine the current-density dependence of the THE at various current densities while keeping the same temperature. To overcome this hurdle, we applied additional cryostat cooling to compensate for the temperature increase due to Joule heating. Initially, at 20 μA with negligible Joule heating, the actual device temperature was determined by the nominal cryostat temperature. The four-probe resistance of the device at 20 μA served as the temperature standard. At higher currents, Joule heating effect led to the increase of local temperature and hence the resistance. We lowered the cryostat nominal temperature to cool down the device and decrease the device resistance to the standard value at 20 μA. Following such strategies, we paired current values with nominal cryostat temperatures one by one so that temperatures at higher currents are calibrated to the same value at 20 μA (see details in Supplementary Fig. 3 and Supplementary Note 1). Therefore, THE measurements are accessible at the same temperature but different currents to investigate the current-density dependence of the THE. Supplementary Fig. 3 shows the temperature compensation chart in which pairs of applied currents and nominal cryostat temperatures correspond to the same resistance and hence the same temperature.

**Figure 4:**
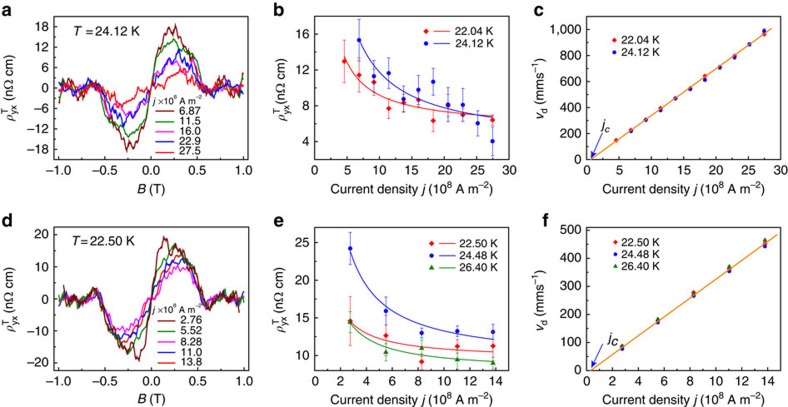
Topological Hall resistivity of MnSi NWs as a function of current density. (**a**) 
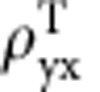
 versus *B* at *T*=24.12 K and (**b**) 
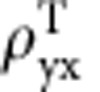
 versus *j* at *T*=22.04 and 24.12 K under increasing current densities from *j*=6.87 × 10^8^ to 27.5 × 10^8^ A m^−2^ for device NW1 (330 nm wide by 270 nm thick). Solids lines represent nonlinear fitting based on equation (4). (**c**) Estimated skyrmion drift velocity (*v*_d_) as a function of current density (*j*). The value and error of each data point are the mean and s.d. of 
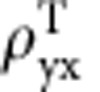
 from 0.2 to 0.4 T at each current density and temperature, respectively. (**d**) 
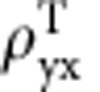
 versus *B* at *T*=22.50 K and (**e**) 
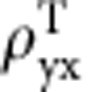
 versus *j* at *T*=22.50, 24.48 and 26.40 K under increasing current densities from *j*=2.76 × 10^8^ to 13.8 × 10^8^ A m^−2^ for device NW2 (480 nm wide by 300 nm thick). Solids lines represent nonlinear fitting based on equation (4). (**f**) Estimated skyrmion drift velocity (*v*_d_) as a function of current density (*j*).

Figure 4a presents topological Hall resistivity at various current densities (*j*) of device NW1 at *T*=24.04 and 22.12 K. The average topological Hall resistivity from 0.2 to 0.4 T versus current density (*j*) is shown in Fig. 4b. The similar set of data for device NW2 are presented in Fig. 4d,e. It is clear that the THE decreases with increasing current densities from 10^8^ to 10^9^ A m^−2^ and then 
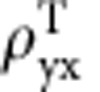
 appears to show a plateau below about 10 nΩ cm. These observations are consistent with the movement of skyrmion domains driven by electrical current and the theoretical prediction that the emergent electric field induced by motion of skyrmions suppresses the THE, as we will discuss in detail below.

## Discussion

To explain the observed current density-dependent THE, we will begin by providing a brief introduction to skyrmion dynamics. The spin-polarized current exerts a force on the underlying magnetic spin structure through the spin-transfer-torque mechanism, a phenomenon that has been explored extensively in ferromagnetic systems^13^,^36^,^37^. At sufficiently high current densities this force is large enough to overcome the pinning forces of the skyrmion domains, and the skyrmion domains begin to translate, as has been observed by the neutron diffraction in bulk MnSi crystals^9^ and LTEM in FeGe thin films^10^. In analogy with Faraday's law, the motion of the effective magnetic field is expected to cause an ‘emergent' electric field perpendicular to the motion of the skyrmions 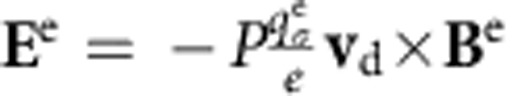 where **B**^**e**^ is the emergent magnetic field associated with a skyrmion, **v**_d_ is the drift velocity of skyrmions. This emergent electric field opposes the topological Hall field arising from the Berry phase of the stationary skyrmions^2^,^25^, leading to a reduction of the measured 
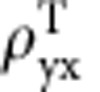
. So the net transverse topological electrical field 
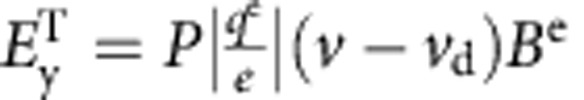
 depends on the relative drift velocity between skyrmions and charge carriers, where *v* is charge carrier drift velocity. Then the current density-dependent topological Hall resistivity can be expressed as^11^


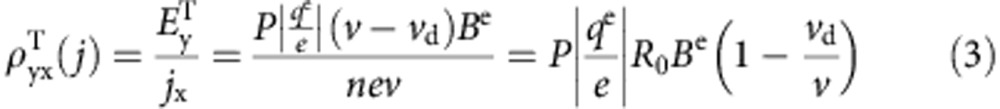


Above the critical current density *j*_c_ (and the critical charge carrier drift velocity *v*_c_), the skyrmion drift velocity (*v*_d_) is linearly proportional to carrier drift velocity (*v*), as suggested by the theory^13^,^25^ and confirmed by experiment^11^. Here we can define a relation *v*_d_=*A*(*v*−*v*_c_) by introducing coefficient *A*. Together with *v*=*jR*_0_, equation (3) turns into


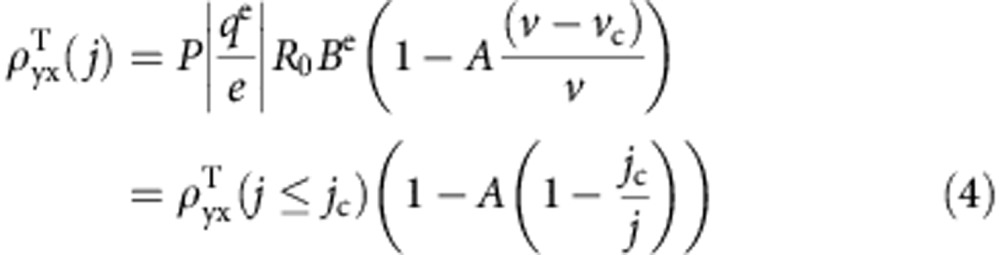


Equation (4) shows how the topological Hall resistivity evolves with the applied current density. At or below *j*_c_, 
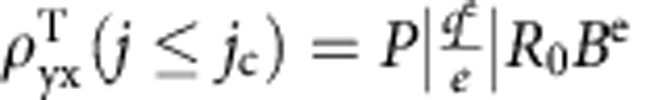
 is the topological Hall resistivity for stationary skyrmions. Slightly or moderately above *j*_c_ where skyrmions start to move, 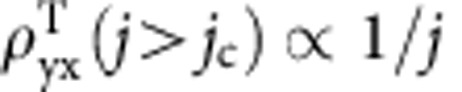. Far above *j*_c_ where 1/*j* approaches zero, 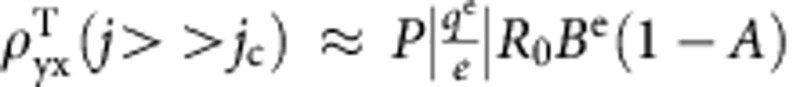, which indicates 
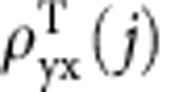
 saturates to the value with a factor (1−*A*) lower than that for stationary skyrmions, which is also predicted in ref. 25. Equation (4) also provides the guideline for searching skyrmion dynamics via observation of the current density-dependent THE. The appropriate observation window should start right around the critical current density *j*_c_ and should not exceed the current density at which the topological Hall resistivity starts to saturate.

The data in Fig. 4b,e agree well with the relation 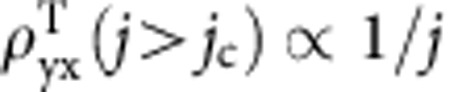, indicating the dynamics of skyrmions in MnSi NWs are in a current regime moderately above *j*_c_. We can estimate the parameters in equation (4) in MnSi NWs as following: current polarization *P* is estimated to be 0.2 (ref. 32), emergent magnetic field is taken as *B*^e^=13.1 T|*e*/*q*^e^|, and normal Hall coefficients were measured to be *R*_0_=32.5 and 37.1 nΩ cm T^−1^ for NW1 and NW2, respectively. Therefore 
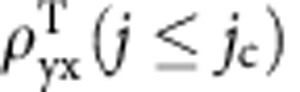
 is estimated to be 85.2 and 97.2 nΩ cm for NW1 and NW2, respectively. Using equation (4) to fit the 
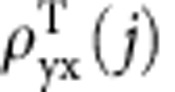
 versus *j* data (Fig. 4b,e) can then allow us to extract fitting parameters *A* and *j*_c_. Coefficients (*A*) and critical currents (*j*_c_) are obtained and averaged across different temperatures to yield *A*=0.95, *j*_c_=7.2 × 10^7^ A m^−2^ for NW1, and *A*=0.91, *j*_c_=2.7 × 10^7^ A m^−2^ for NW2. These critical current densities on the order of 10^7^ A m^−2^ are higher than the ∼10^6^ A m^−2^ observed in MnSi bulk. The possible explanation could be that skyrmions tend to be deflected to one side wall of the NW due to skyrmion Hall effect^2^ so that skyrmions are more easily exposed to surface defects in the NW with high aspect ratio, which could result in the increase of pinning force of skyrmions. Such defects and impurities in the real complex NW samples might also cause slight deviation from the ideal theoretical *A* value^13^.

We then calculate and fit the skyrmion drift velocity (*v*_d_) versus *j* for NW1 (Fig. 4c) and NW2 (Fig. 4f) using:


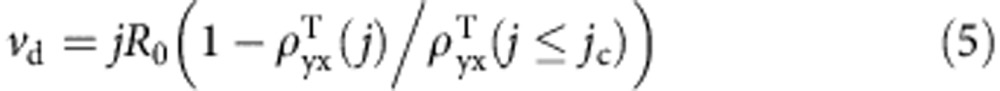


At *j*∼10^9^ A m^−2^, *v*_d_ is ∼330 mms^−1^ for both NW1 and NW2. For each NW device, the *v*_d_ for all temperatures follows one common linear dependence on *j* as indicated by the orange solid lines in Fig. 4c,f. The slopes of these lines are proportional to coefficients *A*=Δ*v*_d_/Δ*v* (0.95 for NW1 and 0.91 for NW2). This means that the skyrmion drift velocity (*v*_d_) increases at a rate slower than the carrier drift velocity (*v*) with a factor of *A*. The behaviour of skyrmion drift velocity is in accord with spin-transfer-torque mechanism^13^,^25^. Our results here uniquely show the skyrmion dynamics arising from the emergent electric field due to the motion of current-driven skyrmions at relatively large current densities in an extended skyrmion stability region that is beyond what has been observed in bulk MnSi samples and its interplay with the THE in NWs.

In summary, we have measured the THE in MnSi NW devices under large current densities, which first shows that the inferred skyrmion phase is stabilized over an extended magnetic field (*B*)-temperature (*T*) window in MnSi NWs compared with bulk MnSi crystals. Furthermore, over a wider temperature window than for bulk materials, we show THE decreases with increasing current density, indicating that the skyrmions are moving due to the electrical current and the THE is suppressed by the emergent electric field arising from motion of skyrmions. The extended skyrmion stability in MnSi NWs and the corresponding current-driven skyrmion dynamics at large current densities are interesting for the fundamental study of the skyrmion physics in confined and/or anisotropic systems^38^, and more importantly demonstrate the feasibility and advantages of exploiting NW systems for potentially using skyrmions in magnetic memory storage systems.

## Methods

### Device fabrication

Electrical measurements were implemented on MnSi NWs obtained via a chemical vapour deposition synthesis^27^. Classical Hall bars were fabricated on MnSi NWs using Ti/Au electrodes defined by e-beam lithography for ohmic electrical contacts. A three-step electrode deposition was employed as illustrated in Supplementary Fig. 1, 20 nm Ti and 20 nm Au was evaporated vertically (the device chip is perpendicular to evaporation direction, see Supplementary Fig. 1a) at first, followed by 60 nm Ti and 30 nm Au at a tilted angle of 45° exposing one side wall of NWs (Supplementary Fig. 1b). The naturally faceted MnSi NWs serve as the shadow masks to ensure there is no contact on the other side of the NWs. Then the device chip was remounted and 90 nm Ti and 40 nm Au was evaporated at a tilted angle of 45° exposing the opposite side wall of NWs (Supplementary Fig. 1c). Hall measurements were made in which the outermost electrical contacts act as current source and drain, and the inner two contacts contacting opposite sides of the facetted MnSi NWs allow detection of the transverse Hall voltage (see Fig. 1a inset for a SEM image of a typical MnSi NW Hall device).

### Device measurements

The devices were measured in a Quantum Design PPMS-9T to provide the temperature and magnetic field control, with the DC current supplied by a Keithley 6221 current source and the voltage across the transverse contacts monitored by a Keithley 2182A nanovoltmeter. The MnSi NWs were tested at room temperature for ohmic contact and were cooled from 300 K down to 10 K at a rate of 5 K min^−1^ under zero applied magnetic field. Once base temperature was reached, the device was allowed to equilibrate at 10 K for 30 min, and the field was set to −1 T. After collecting data across the field range from −1 to +1 T the data collection was repeated in the reverse direction (from +1 to −1 T) to check the reproducibility of the observed signals as well as to detect any magnetic hysteresis. After completion of the data collection to the final field of the second scan, the field was held at the final value of −1 T and the temperature was set to the next value at a rate of 2 K min^−1^, and the device was allowed to equilibrate at the new set temperature for 20 min before data collection. The 20 min equilibration time was found to be more than sufficient to reduce any artifacts caused by thermal equilibration to a negligible level.

## Additional information

**How to cite this article:** Liang, D. *et al*. Current-driven dynamics of skyrmions stabilized in MnSi nanowires revealed by topological Hall effect. *Nat. Commun.* 6:8217 doi: 10.1038/ncomms9217 (2015).

## Supplementary Material

Supplementary Information Supplementary Figures 1-3 and Supplementary Note 1
