# Supplementary material for: Current-driven dynamics of skyrmions stabilized in MnSi nanowires revealed by topological Hall effect
Source: Nat Commun. 2015 Sep 24;6:8217. doi: 10.1038/ncomms9217 (PMC4598358; doi:10.1038/ncomms9217)
Supplement: Supplementary Information — Supplementary Figures 1-3 and Supplementary Note 1 [file ncomms9217-s1.pdf]

Supplementary Figures

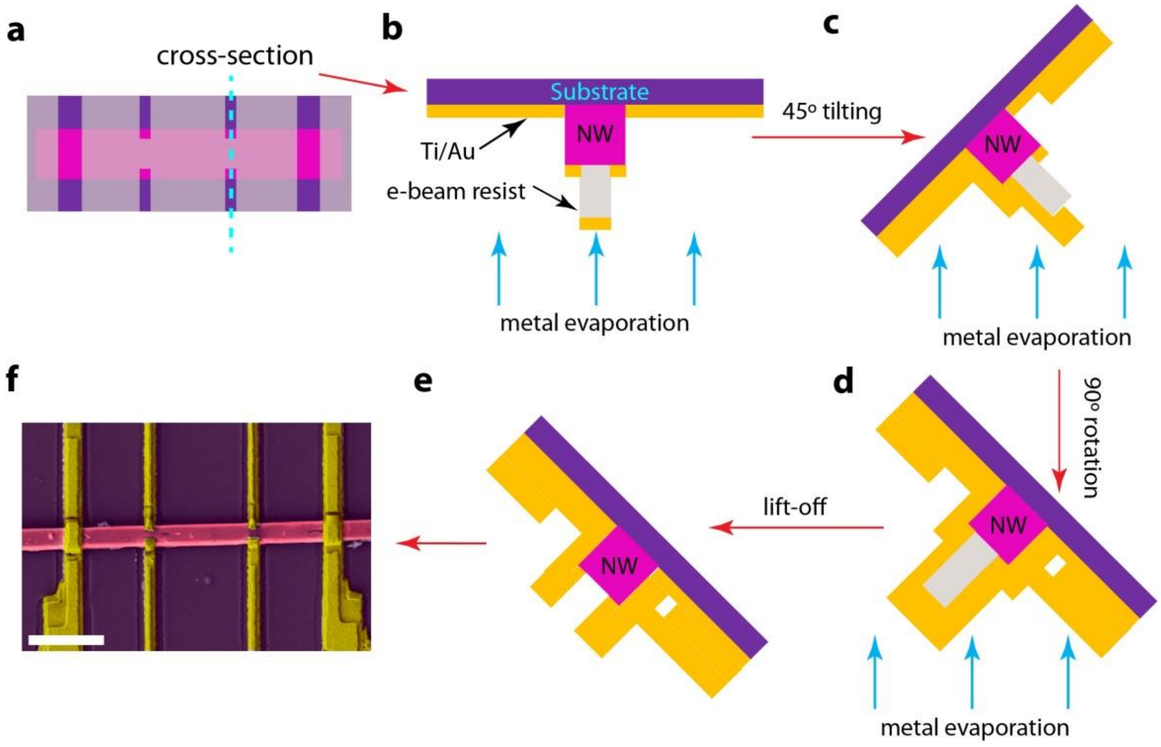

Supplementary Figure 1 | Schematic fabrication procedures of MnSi NW Hall bar device. (**a**) The bird-eye schematic view of a six-probe Hall bar pattern on a MnSi NW after e-beam resist is developed but before the metal is deposited. The purple, pink, gray colors represent the substrate, the NW, and the e-beam resist, respectively. The cyan dash line highlights the cross-section of one pair of Hall bars for illustration of metal evaporations in panels **b-e**. (**b**) The cross-sectional view of vertical metal evaporation of a 20/20 nm Ti/Au layer shown by the golden color. (**c**) Schematic view of a 45° angled metal evaporation of the second layer (60/30 nm Ti/Au) to contact one side wall of the NW. (**d**) Schematic view of the 45° angled evaporation of the third layer (90/40 nm Ti/Au) to contact the other side wall. (**e**) Schematic cross-sectional view of the NW device after lift-off in acetone. (**f**) A typical SEM image of a MnSi NW with six-probe classical Hall bars. Scale bar is 2 μm.

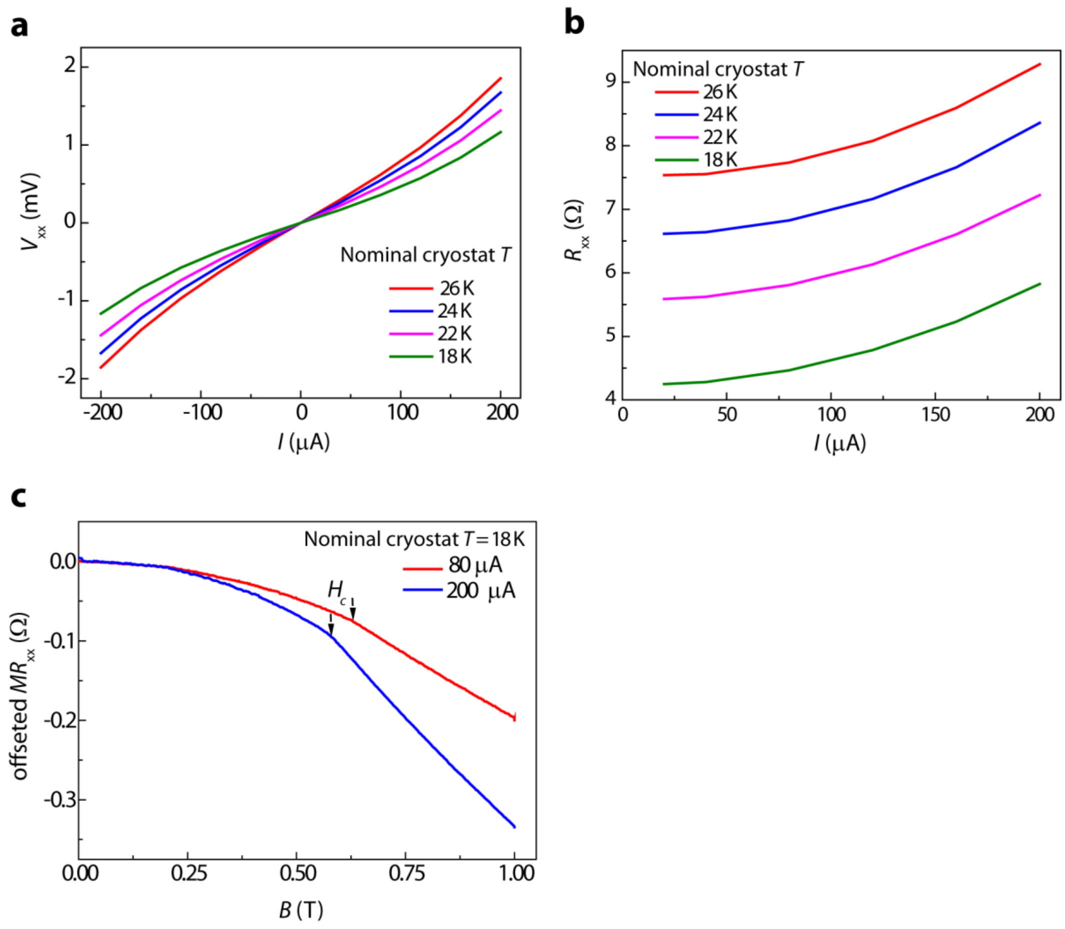

Supplementary Figure 2 | Joule heating effect of MnSi NW devices. (**a**) Longitudinal voltage (V_{xx}) vs. current (I) at nominal cryostat temperatures from 18 K to 26 K. (**b**) The corresponding differential resistance (R_{xx}) vs. current (I) from nominal temperature18 K to 26 K. The increases of R_{xx} with T indicate Joule heating. (**c**) Offset MR at I = 80 μA and 200 μA for nominal cryotat T = 18 K. Joule heating is revealed by the shift of the critical field (H_c).

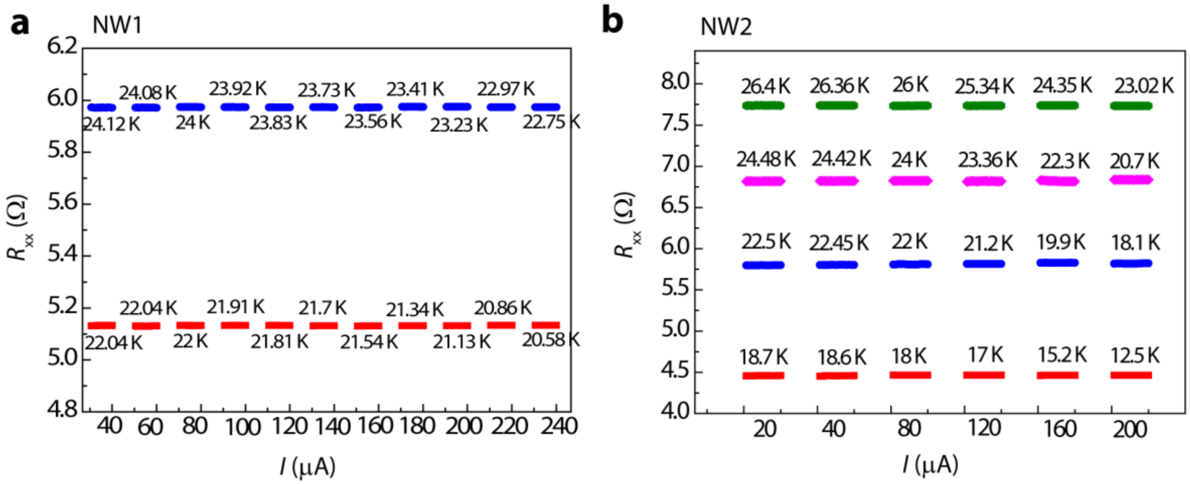

Supplementary Figure 3 | Combination pairs of cryostat temperatures and applied currents corresponding to the same NW device resistance at each temperature for (a) NW1 and (b) NW2, for Joule heating compensation. The device temperature was determined by the nominal cryostat temperature at 20 μA. The resistances were calibrated to the values at 20 μA for all the combination pairs of nominal cryostat temperatures and applied currents, which indicates the device reaches the same actual temperature at all currents.

Supplementary Note 1

Joule heating effect and temperature compensation of MnSi NW devices. Before we studied the current density dependence of topological Hall effect (THE), we need to make sure the device temperature is not affected by the current, since applying large currents to nanodevices will likely introduce Joule heating. We first took the I-V_{xx} curve to check if Joule heating appears. As shown in Supplementary Fig. 2a, we observed nonohmicity of I-V. Supplementary Fig. 2b shows the current dependence of differential resistance $R_{xx} = dV_{xx}/dI$ for a typical MnSi NW device. The resistance is supposed to be constant if no Joule heating shows up. However, the resistance increases with increasing current. Together with the fact that resistance of a metallic MnSi NW increases with increasing temperature, we conclude that the increase of resistance with current originates from the Joule heating effect of applied current. The devices were usually warmed up by large currents above 40 μA and the resistance increased more as currents increased further to result in more heating. (Note that NWs were not examined in an electron microscope prior to measurements, the exact current density applied to the device can only be calculated after the electrical measurements were complete.)

Another experimental evidence to confirm Joule heating is the shift of the critical field B_c in the longitudinal magnetoresistance (*MR*) of NW devices in a perpendicular field as shown in Supplementary Fig. 2c. At nominal T = 18 K of Physical Properties Measurement System (PPMS), the critical field H_c, which corresponds to the transition field from conical to field-polarized ferromagnetic states, shifted from 0.63 T for *MR* at 80 μA to 0.58 T for the *MR* at 200 μA, indicates the warming up of the device by the current since the critical field H_c was shown to decrease with the increasing temperature.

Studying the current density dependence of the topological Hall effect requires that all measurements at different currents are taken at the same temperature. In order to overcome the Joule heating problem and fulfill this requirement, we employed PPMS cryostat cooling to compensate for the Joule heating of the NW devices. First of all, the actual device temperature at I = 20 μA was determined by the nominal temperature of the cryostat. The 4-probe resistance R_{xx} of the NW device served as the temperature standard. When we applied currents larger than 20 μA, the local temperature of NW device increased as well as the resistance. Here, we applied cooling by lowering the nominal cryostat temperature to make sure the device resistance went back to the initial value at 20 μA. In such a way, the actual device temperatures at high currents were calibrated to the temperature at 20 μA. . As a result, applied currents and nominal cryostat temperatures formed pairs corresponding to the same temperature. Therefore, the actual NW device temperatures are the same at different currents after compensation. For example, we used R_{xx} = 7.735 Ω at cryostat T = 26.4 K, I = 20 μA as the standard. While we apply larger currents of 40 μA, 80 μA, 120 μA, 160 μA, and 200 μA, the resistances increase as shown by Supplementary Fig. 2b. In order to reach the initial R_{xx}, we decreased the cryostat temperature to compensate the Joule heating of the device by setting cryostat T = 26.36 K, 26 K, 25.34 K, 24.35 K, 23.02 K, respectively. Finally, the resistances reach the same value for pairs of 20 μA and 26.4 K, 40 μA and 26.36 K, 80 μA and 26 K, 120 μA and 25.34 K, 160 μA and 24.35 K, 200 μA and 23.02 K.

In Supplementary Fig. 3, we show the combination pairs of nominal cryostat temperatures and applied currents corresponding to the same resistance value at each temperature for NW1 and NW2 discussed in the main text. The temperatures in the figure represent nominal cryostat temperatures.
